# Differences in tuberculosis prevalence by sex in low- and middle-income countries over 1993–2025: A systematic review and meta-analysis

**DOI:** 10.1371/journal.pmed.1005114

**Published:** 2026-05-22

**Authors:** Nicole A. Swartwood, Nanki Singh, Seyed Alireza Mortazavi, Melike Hazal Can, Hening Cui, Do Kyung Ryuk, Peter MacPherson, Katherine C. Horton, Nicolas A. Menzies

**Affiliations:** 1 Department of Global Health and Population, Harvard T H Chan School of Public Health, Boston, Massachusetts, United States of America; 2 Grossman School of Medicine, New York University, New York City, New York, United States of America; 3 School of Health and Wellbeing, University of Glasgow, Glasgow, United Kingdom; 4 Department of Infectious Disease Epidemiology, London School of Hygiene and Tropical Medicine, London, United Kingdom; 5 Center for Health Decision Science, Harvard T H Chan School of Public Health, Boston, Massachusetts, United States of America; Duke University, UNITED STATES OF AMERICA

## Abstract

**Background:**

Global and national initiatives to combat tuberculosis (TB) have expanded over recent years. Despite this, the TB burden remains high in some population groups, with men recognized as having elevated TB risks. Summary measures of sex differences in TB prevalence were last estimated in 2016. Since then, many additional prevalence surveys have been conducted, including in the highest TB burden countries. We conducted a systematic review of sex-stratified TB prevalence survey data published over 1993–2025, to provide updated estimates of male-to-female (M:F) TB prevalence ratios and determine whether sex-related disparities in TB burden have closed over time.

**Methods and findings:**

We identified surveys reporting community-representative, sex-stratified estimates of pulmonary TB prevalence in low- and middle-income countries (LMICs), including surveys from an earlier review (covering January 1993–March 2016) and a new systematic review (covering 1st December 2015–13th October 2025). This review was prospectively registered with PROSPERO (CRD42024503853) and included searches of PubMed, Embase, Global Health, the Cochrane Library, Africa Index Medicus, LILACS, and SciELO. We extracted data on bacteriologically confirmed and smear-positive TB prevalence among adults (aged ≥ 15 years), stratified by sex. Risk of bias was evaluated using eight criteria specific to prevalence surveys. We fit multi-level Bayesian regression models with study- and country-level random effects to estimate the M:F ratio of TB prevalence (male prevalence divided by female prevalence), overall and for key subgroups. In meta-regression analyses, we estimated how prevalence ratios varied over time and according to known TB risk factors and TB case definitions.

We identified 10,124 publications and extracted data from 100 eligible studies representing 102 unique prevalence surveys and 4,658,310 participants (45.6% male) in 33 LMICs. TB prevalence was higher in men than women in 90/102 of the included surveys, with a pooled M:F prevalence ratio of 2.02 (95% credible interval (CrI): 1.71, 2.34) for bacteriologically confirmed TB and 2.38 (95% CrI: 1.91, 2.90) for smear-positive TB. Time trend analyses showed a 2.0% (95% CrI: −0.2, 4.5%) average annual change in the M:F ratio of bacteriologically confirmed TB over the study period. The M:F prevalence ratio was estimated to be higher for countries with greater excess HIV prevalence among men, and countries with greater gender equity (as measured by the United Nation’s Gender Development Index). The estimated M:F prevalence ratio was also higher for surveys that did not restrict testing to individuals reporting TB symptoms. Study limitations include heterogeneity in survey methods and definitions, as well as limited data from the Americas, Eastern Mediterranean, and Europe WHO world regions and post-COVID-19 period.

**Conclusions:**

Men in LMICs consistently experience TB at a higher prevalence than women. Time trend estimates are uncertain, but consistent with widening sex differences in TB prevalence over the last three decades, despite efforts to address the risk factors underlying this excess TB burden.

## Author summary

**Why was the study done?:**

- Previous studies, including a 2016 systematic review and meta-analysis, have identified substantial sex differences in tuberculosis (TB) burden, with higher TB infection, prevalence, and mortality consistently observed among men in low- and middle-income countries.
- The change in these sex differences over time has not been previously estimated. Recent improvements in TB case detection efforts, diagnostics, and treatment approaches may differentially affect TB epidemiology among men and women. Additionally, newly conducted TB prevalence surveys have increased the duration and diversity of evidence to inform trends in TB burden.

**What did the researchers do and find?:**

- We systematically identified 102 national and sub-national TB prevalence surveys conducted in low- and middle-income countries and used Bayesian meta-regression models to estimate male-to-female TB prevalence risk ratios overall and among key demographic and epidemiological subgroups. We also estimated how prevalence risk ratios varied according to known TB risk factors, TB case definitions, and over time (1994–2024).
- Adult men have over twice the prevalence of pulmonary TB as compared to women in low- and middle-income countries (LMICs). Increased prevalence was associated with greater excess HIV prevalence among men, and countries with greater gender equity (as measured by the United Nation’s Gender Development Index). The estimated M:F prevalence ratio was higher among surveys that did not restrict testing to individuals reporting TB symptoms.
- Temporal analysis suggested that male-to-female inequalities in TB prevalence might be growing, particularly in the World Health Organization (WHO) Africa region.

**What do these findings mean?:**

- Despite global commitments to gender equity in health, men in LMICs continue to bear a disproportionate burden of TB.
- If sex-related inequalities in TB burden are growing, developing effective strategies to reduce men’s risk of TB and to engage men in TB prevention and care will be essential to end TB.
- Limitations include limited prevalence survey data in the Americas, Eastern Mediterranean, and Europe WHO world regions, methodological heterogeneity across surveys, especially among subnational surveys, and few prevalence surveys from the post-COVID-19 period.

## Introduction

Despite sustained global efforts to reduce tuberculosis (TB), it remains the leading cause of death from a single infectious agent globally [1]. In 2024, men were estimated to account for approximately 60% of TB incidence among adults and TB deaths among HIV–negative adults worldwide [1]. The mechanisms underpinning these differences, and the extent to which they vary across time and settings, are not well understood.

Gender—defined as the set of socially constructed roles, behaviors, and norms related to perceived sex—is a key determinant of health and shapes TB susceptibility, exposure risks, engagement with prevention and care, and outcomes [2–5]. Gendered health behaviors and systemic healthcare access barriers can lead to delayed diagnosis and worse care engagement among men, contributing to ongoing transmission and under-detection. In addition, sex-assortative mixing among men—particularly in workplaces, social settings, and high-risk congretate settings such as prisons or homeless shelters—can amplify transmission within male networks [2,3]. Men also have a higher prevalence of risk factors that increase both susceptibility to infection (e.g., tobacco use, occupational, and ambient air pollution) and progression from infection to TB disease (e.g., untreated HIV, diabetes, malnutrition, and smoking). Together, sex and gender interact with structural determinants, such as occupational exposures, unequal health system access, and prevailing societal norms, to produce a persistent excess burden of TB among men [4,5]. Conventionally, measures of disease burden are reported by biological sex, not gender. However, given the considerable overlap of sex and gender identities within populations, sex-stratified outcomes will reflect the joint impact of these two related, but distinct, factors.

While routinely reported, TB case notifications have marked limitations as measures of TB burden, as they can be affected by incomplete case detection and reporting. National TB prevalence surveys are designed to generate population-representative estimates of TB burden unaffected by these potential biases and have been conducted in a number of high-burden countries with the support of the World Health Organization (WHO). Historically, these surveys have reported higher TB prevalence in men than in women in low- and middle-income countries (LMICs) [6]. A 2016 systematic review and meta-analysis by Horton and colleagues estimated that men in LMICs had more than twice the prevalence of TB compared with women [7].

TB epidemiology is not static and will change under the influence of multiple factors. During the COVID-19 pandemic, many TB resources were reallocated to pandemic response—an effort that led to the pronounced decreases in TB case detection and increases in TB deaths [8,9]. Most recently, global and national commitments to TB elimination have been renewed through accelerated case detection and introduction of new diagnostics and treatment approaches [10]. There has also been a scale-up of interventions to address key risk factors for TB, including increased treatment of HIV [11]. Alongside these changes, a substantial number of new prevalence surveys have been conducted since 2016, including in some of the world’s highest TB burden countries (e.g., India, South Africa) [1]. Sex differences in TB prevalence might have changed in response to these factors, or as a result of the increasing recognition of sex as a TB risk factor [1,12], most notably the addition of men to WHO’s list of populations vulnerable to TB [13]. However, empirical analyses have not described whether sex differences in TB burden have changed over time.

In this study, we undertook a systematic review and meta-analysis of sex-disaggregated TB prevalence surveys conducted in LMICs, published between 1st January 1993 and 13th October 2025. Based on this review, we investigated whether sex differences in TB prevalence (operationalized by the male-to-female ratio of TB prevalence) have changed over time. We also explored how this ratio is correlated with key socio-demographic determinants and approaches used to determine TB prevalence.

**Table 1:** Multiplicative effect of standardized^3^ covariates on male-to-female ratio of bacteriologically-confirmed TB prevalence estimated from nationally representative surveys (*N* = 38)^4.^

	Univariable			Multivariable		
	Posterior mean	Posterior median	95% credible interval	Posterior mean	Posterior median	95% credible interval
Intercept	2.23	2.23	1.96, 2.52	2.25	2.25	2.00, 2.55
UN Gender Development Index	1.15	1.15	1.03, 1.28	1.16	1.16	1.00, 1.34
Study end year	1.01	1.01	0.99, 1.03	1.01	1.01	0.99, 1.03
Absolute difference in male-to-female prevalence of:						
Alcohol use disorder^1^	1.08	1.08	0.95, 1.23	1.01	1.01	0.89, 1.15
Type II Diabetes^1^	0.95	0.95	0.83, 1.08	1.00	1.01	0.87, 1.14
HIV/AIDS^1^	1.12	1.12	1.00, 1.26	1.18	1.18	1.03, 1.35
Smoking^2^	1.09	1.09	0.96, 1.24	1.05	1.05	0.92, 1.19
Underweight (BMI < 18.5)^2^	0.94	0.94	0.83, 1.07	1.01	1.01	0.88, 1.17

Notes: UN, United Nations; BMI, body mass index.

^1^Estimated from Global Burden of Disease Study 1994–2023 (GBD 1994–2023) results.

^2^Estimated from Global Burden of Disease Study Covariates 1994–2023.

^3^All covariates, except study end year, were standardized to a mean of zero and unit standard deviation. The standard deviations of the unscaled covariates are Gender Development Index = 0.062; Alcohol use disorder = 1368.932 (per 100,000); Type II Diabetes = 647.394 (per 100,000); HIV/AIDS = 1966.388 (per 100,000); Smoking = 2.30 (per 100); Underweight = 0.040 (per 100). The study end year was mean-centered, and the coefficients represent the multiplicative effect for one additional year.

^4^UN Gender Development Index estimates are not produced for the Democratic People’s Republic of Korea and Eritrea; the nationally-representative surveys for these two countries were omitted from univariable and multivariable analysis.

## Methods

We conducted a systematic review and meta-analysis of sex-stratified TB prevalence surveys conducted among people living in LMICs (as defined by the World Bank) [14], We registered the review and meta-analysis with the International Prospective Register of Systematic Reviews (PROSPERO) protocol number CRD42024503853 [15]. We followed the Meta-analysis of Observational Studies in Epidemiology (MOOSE) guidelines for the conduct of the review (S1 Checklist) and the Preferred Reporting Items for Systematic Reviews and Meta-analyses (PRISMA) guidelines for preparing the manuscript (S2 Checklist) [16,17]. We used the Covidence online systematic review tool to manage the review [18].

### Search strategy

We designed the search strategy as an update of a previous systematic review and meta-analysis by Horton and colleagues [7]. Our search included a combination of MeSH terms and keywords specifically related to “tuberculosis,” “prevalence,” and “low- and middle-income countries (LMICs).” We searched four electronic databases—Global Health, the Cochrane Database of Systematic Reviews, PubMed, and Embase—between 1st December 2015 and 13th October 2025 for English language publications. The full search strategy is reported in Table A in S1 Appendix. We additionally evaluated all studies included in the previous analysis for inclusion [7] and compared our included studies against the national TB prevalence surveys listed in WHO Global Tuberculosis Report 2025, adding any survey reports that were not captured by our search [1]. We also examined the citations of all identified publications for additional relevant material. Finally, we screened published abstracts from the 2023 Union World Conference on Lung Health for recent prevalence surveys [19].

### Inclusion and exclusion criteria

We reviewed all articles and included TB prevalence surveys that reported sex-stratified estimates of adult (aged ≥ 15 years) pulmonary TB prevalence from a population-based sample, conducted in a LMIC (based on 2024 World Bank classification). Extra-pulmonary TB was excluded due to the diagnostic complexity of this condition, which often necessitates invasive diagnostic procedures not typically available in community survey settings. We also excluded studies that only evaluated care-seeking persons, surveys solely reliant on self-reported data without laboratory confirmation, surveys conducted following a community-based active case finding intervention in the same setting (as the intervention may have altered local TB prevalence), and surveys of children aged 14 years or younger, as determining TB status can be challenging in this group. Detailed inclusion and exclusion criteria can be found in Tables B and C in S1 Appendix.

Our review team consisted of nine people (NS, NAS, AM, MHC, HC, DKR, PM, KCH, and NAM). Two reviewers (of NS, NAS, AM, MHC, HC, and DKR, allocated at random) independently assessed the titles and abstracts of all studies to identify those requiring full text review. Discrepancies were resolved through consensus discussions, mediated by a third reviewer (NAS, PM, KCH, or NAM). Two reviewers (of NS, NAS, AM, MHC, HC, DKR, PM, and KCH) independently evaluated full texts for study inclusion, with discrepancies being resolved through discussion as outlined above.

### Data extraction

Two reviewers independently extracted data on study methodology, risk of bias, and TB prevalence using a pretested extraction form (S1 Form). Discrepancies between reviewers were resolved by a third reviewer. Where multiple studies reported on the same TB prevalence survey, we merged the studies and extracted all available data; where conflicting data were reported, we extracted data from the most recent publication. Where studies reported multiple prevalence surveys, data were extracted separately for each survey. Therefore, the unit of analysis in our meta-analysis was the prevalence survey as opposed to the publication.

### Risk of bias

We assessed the risk of bias for each included study using eight criteria adapted from the Hoy and colleagues tool for the appraisal of prevalence surveys [20]. This tool examines study population selection, nonresponse bias, data collection methodology, and case definition parameters. The assessment criteria are listed in the extraction form (S1 Form, Section 8). Reviewers were also asked to provide an overall assessment of risk of bias (“low”, “moderate”, or “high”) based on the eight criteria.

To evaluate the robustness of our primary English-language search strategy, we also searched three databases—Africa Index Medicus, LILACS, and SciELO—for French, Portuguese, and Spanish publications over the full study period (1st January 2009–13th October 2025). The corresponding search strings are in Table D in S1 Appendix. Non-English titles and abstracts were translated to English using DeepL, a neural machine translation (NMT) tool; studies identified for full-text review were similarly translated.

### Definitions

To be eligible for quantitative analysis, a survey was required to report a measure of bacteriologically confirmed TB. Bacteriologically confirmed TB was defined as TB confirmed through bacteriological methods, requiring at least one positive result of smear microscopy, culture, or a molecular diagnostic (e.g., Xpert MTB/RIF). Where reported, we adopted the study definition of bacteriologically confirmed TB. In the absence of a study definition, we used estimates of culture-positive TB prevalence or smear-positive TB prevalence, prioritized in that order. Smear-positive TB was defined as TB confirmed through sputum smear microscopy, regardless of other diagnostic results. Where studies reported more than one prevalence measure, adjusted TB prevalence estimates with 95% confidence intervals were the preferred measure used for synthesis, followed by crude prevalence estimates with 95% confidence intervals, then counts of individuals with TB and total number tested, or summary prevalence estimates without a confidence interval (Fig A in S1 Appendix).

Survey year was set to the end year of data collection; where the end year was missing, we set the survey year to the publication year less the mean observed publication delay in our data, which was 4 years.

Participants were classified as male or female by the underlying prevalence review, the basis for which was not commonly reported. As such, we adopted these labels as reported for our analysis.

### Data synthesis and statistical analysis

Reported data from all surveys were converted to prevalence per 100,000 persons. We took one of two approaches to calculate the standard error of each survey-reported prevalence estimate. If confidence intervals were reported, we used these to back-calculate the standard error; for estimates without confidence intervals, we applied the normal approximation method of the binomial standard error. Standard errors were estimated on the log scale. All crude prevalence estimates (i.e., those not already adjusted for features of the survey design) had a variance correction factor applied in order to approximate the additional sampling variance due to complex survey designs.

Our primary outcome was the male-to-female (M:F) ratio of bacteriologically confirmed TB prevalence, calculated as TB prevalence among males divided by TB prevalence among females. We used Bayesian multilevel meta-regression models to synthesize evidence from the pooled survey data, with models constructed for the log of the M:F prevalence ratio. This log transformation was adopted to improve the symmetry and homoscedasticity of residuals for the fitted model. This approach also produced a multiplicative relationship between predictor variables and the M:F prevalence ratio, allowing results to be interpreted as risk ratios. We adopted an identity link function and specified a normal likelihood function for the survey data. The standard deviation of individual observations (the log of study-specific M:F prevalence ratios) was calculated from study-reported standard errors. We chose our priors to be weakly informative, based on published guidance [21], and tested the impact of alternative prior specifications on the study results. To estimate overall and study-specific M:F prevalence ratios, we fit models with study- and country-level random effects. To estimate M:F prevalence ratios for each WHO region, we fit models with study- and region-level random effects. For each model, random effects and associated variance parameters were assigned priors and estimated simultaneously, such that uncertainty in these values was fully propagated into posterior inference. We examined these results to report the relative magnitude of random effect variance at the country (or region) and study level. All model equations and priors are reported in Table E in S1 Appendix.

To explore how the M:F prevalence ratio changed over time, we revised the regression models to include a term for study year and added random effects for the interaction of this time trend with study country or WHO region, respectively. From the results of these models, we calculated the estimated annual percentage change (EAPC) in the M:F ratio of bacteriologically confirmed TB prevalence, overall and by WHO region. We also calculated the probability that these EAPC estimates were positive (P(EAPC > 0)), as an indicator of whether the magnitude of the M:F prevalence ratio was increasing over time.

We extended these regression models to investigate univariable and multivariable country-level exploratory associations between several TB determinants and the M:F ratio of bacteriologically confirmed TB. We included covariates for survey year, United Nations’ Gender Development Index (GDI) [22]. and absolute difference in country-specific male and female levels of prevalence of alcohol use disorder, type II diabetes mellitus, HIV/AIDS, underweight as measured through low body mass index (BMI), and smoking, obtained from the Global Burden of Diseases Project (GBD) [23,24]. Covariates were matched to each survey based on survey country and end year, and standardized globally to a zero mean and unit standard deviation. As such, coefficient estimates reflect the change in the log M:F prevalence ratio associated with a 1 standard deviation change in the predictor. GBD covariates were missing for one survey (2.5%) and GDI was missing for five surveys (12.5%); where available (one of GBD and three of GDI missing surveys), these surveys were assigned the value corresponding to the nearest available year. We list the affected surveys in Table F in S1 Appendix. We fitted these models using data from 38 of 40 nationally-representative surveys (GDI was not available for the Democratic People’s Republic of Korea or Eritrea, and the corresponding surveys were omitted from univariable and multivariable analyses).

We applied the main effect model to subgroups of the overall survey data, such as surveys stratified by survey representativeness (national or subnational), risk of bias (low or not low), use of symptom screening (yes or no), and whether bacteriologically confirmed TB required a positive Xpert MTB/RIF or culture, to explore potential differences in the M:F ratio of bacteriologically confirmed TB prevalence. For countries represented by five or more surveys, we estimated country-specific M:F prevalence ratios. We also examined how our results compared with the Horton and colleagues 2016 review by replicating our main effect model for those only those studies included in the previous study. Finally, as an alternative examination of the change in M:F ratios of bacteriologically confirmed TB prevalence over the study period, we fit the main effect model to extracted data grouped into five-year bands (e.g., 2000–2004, 2005–2009, etc.) by survey end year.

We tested several alternative model specifications. We revised the main effects and time trend models to include binary covariates representing variation in the use of more sensitive TB diagnostics (i.e., Xpert MTB/RIF or culture) in each survey, and whether symptoms were required for sputum collection. We also explored alternative hierarchical structures, including nested country-random effects within WHO region-random effects.

All analyses were conducted in R (version 4.4.2) using the `brms` and `tidybayes` packages [25–27]. The model ran for 10,000 iterations (2,500 burn-in) on 4 chains. Convergence was assessed through inspection of each model’s effective sample size (ESS), potential scale reduction factor (R), and posterior predictive checks. For all outcomes, we report the posterior mean and equal-tailed 95% credible interval (representing an interval with 95% probability of containing the true value) obtained from summarizing 10,000 posterior draws per parameter.

## Results

**Fig 1:**
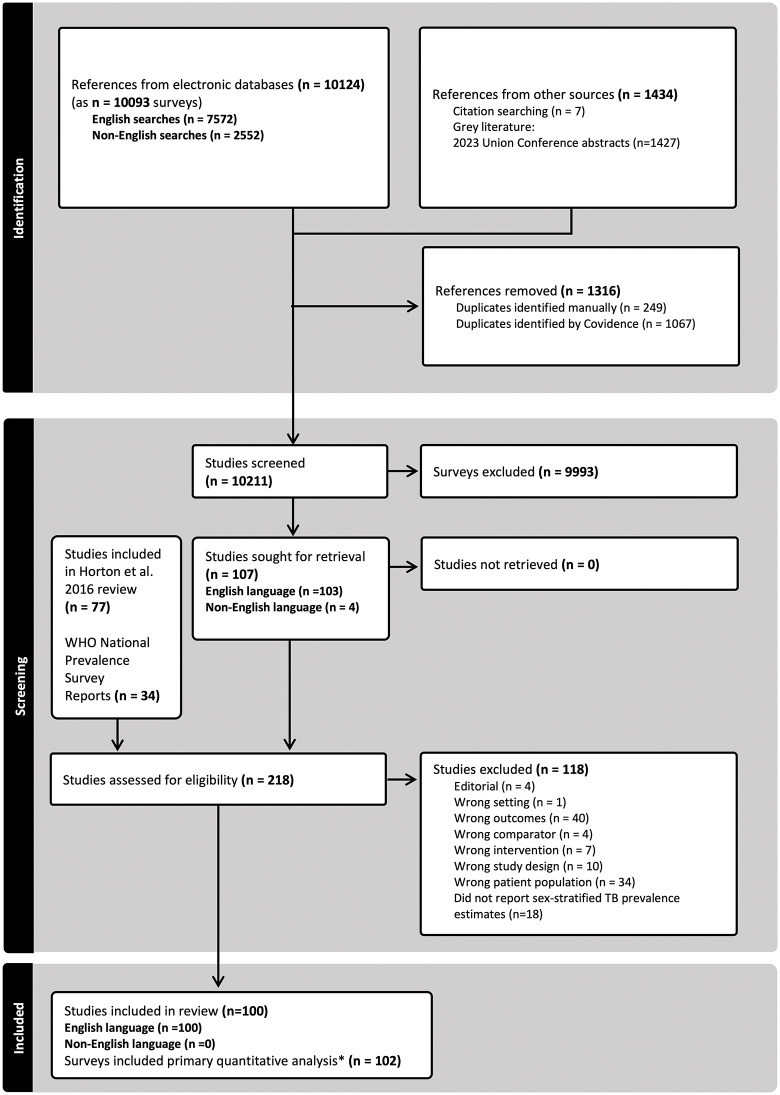
PRISMA diagram of study selection.

**Fig 2:**
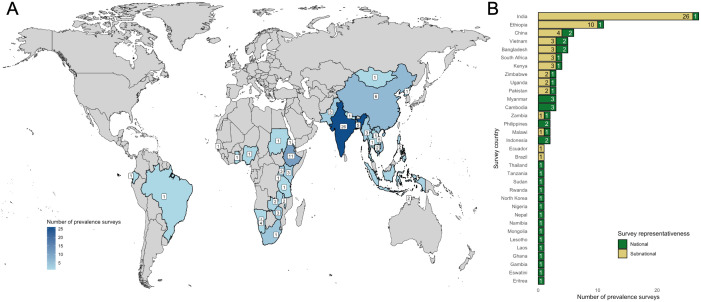
Distribution and counts of included prevalence surveys. Panel **A**: Geographic distribution of surveys, including the total number of surveys in each country. Panel **B**: Number of subnational and national TB prevalence surveys per country. Note: Map was generated using the *maps* R package [156].

Of the 10,124 publications screened by title and abstract, 216 had their full text reviewed (Fig 1). Table G in S1 Appendix lists the publications excluded at the full-text review stage with their corresponding exclusion reasons. The most common exclusion reasons were wrong outcome (39%) and wrong study population (34%). We identified 100 English-language publications that described 102 unique surveys reporting sex-stratified bacteriologically confirmed TB prevalence estimates [28–127]. We did not find any relevant surveys in the examined French, Portuguese, or Spanish studies. The characteristics of included surveys and participants are reported in Tables H and I in S1 Appendix. These surveys were conducted in 33 countries across five WHO world regions: 16 in Africa region, seven in the South-East Asia region, six in the Western Pacific region, two in the Eastern Mediterranean region, and two in the region of the Americas (Fig 2). All included countries were classified as WHO high TB incidence countries. Overall, 100/102 surveys reported total participant numbers, giving 4,658,310 participants; 96/102 surveys reported sex-stratified participant numbers, with 45.6% male participants.

**Fig 3:**
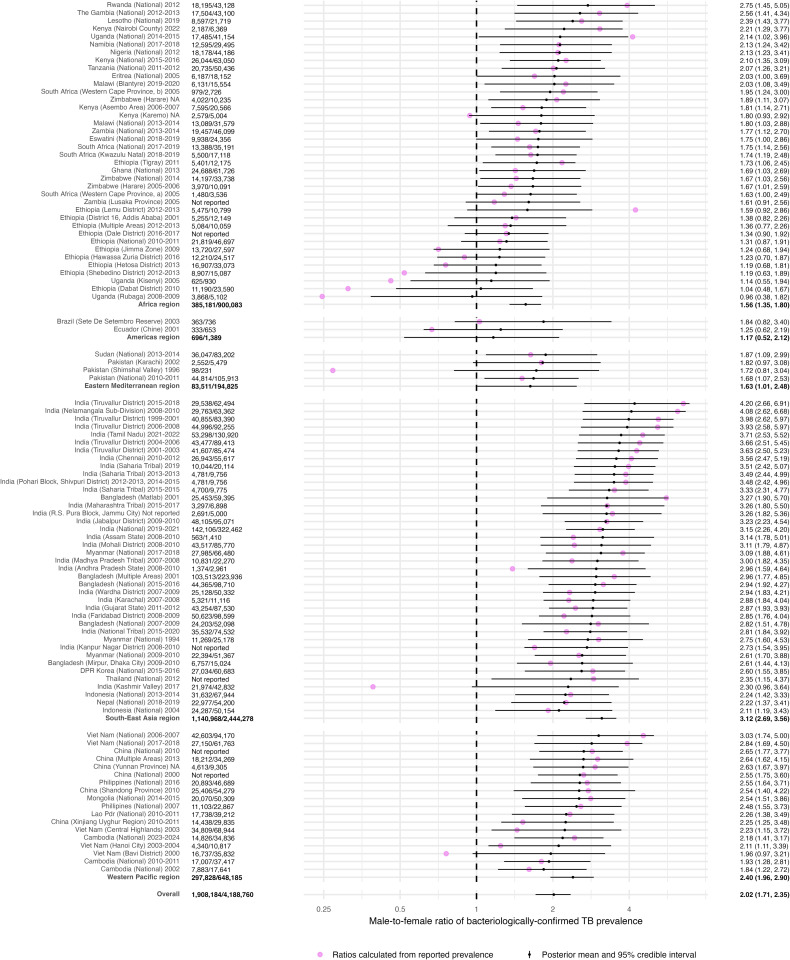
Male-to-female ratios of bacteriologically confirmed (*n* = 102) TB prevalence by world region, as compared with ratios calculated from survey reported data.

Among the identified surveys, 90 (88%) reported greater bacteriologically confirmed TB prevalence among men than women; all of the 40 nationally representative surveys reported greater male bacteriologically confirmed TB prevalence. The overall pooled M:F ratio of bacteriologically confirmed TB was 2.02 (95% credible interval: 1.71, 2.34; Fig 3). Posterior predictive checks did not reveal any major systematic discrepancies between model estimates and the underlying data. Details on model performance can be found in Table J and Fig B in S1 Appendix. Table K in S1 Appendix contains estimated M:F ratios from an alternative model hierarchical structure. We also estimated the impact of requiring symptoms for sputum collection and differential diagnostic algorithms on the male-to-female ratio of bacteriologically confirmed TB prevalence; these estimates are reported in Table L in S1 Appendix. Surveys that required symptoms for sputum collection had lower M:F ratios compared to surveys that did not (multiplicative effect: 0.64; 95% CrI: 0.51, 0.80). Surveys that used Xpert MTB/RIF or sputum culture as a component of the diagnostic cascade had higher M:F ratios compared to surveys that only used sputum smear microscopy (multiplicative effect: 1.48; 95% CrI: 1.12, 1.94).

In each WHO region, bacteriologically confirmed TB prevalence was higher among males compared with females, ranging from 1.76 (95% CrI: 1.17, 2.49) in the Eastern Mediterranean to 2.91 (95% CrI: 2.51, 3.33) in South-East Asia. Posterior summaries indicated that the between-country and between-region variance exceeded within-county or within-region (i.e., survey-level) variance, respectively (Fig C and Table M in S1 Appendix). This reallocation of variance is consistent with country and region random effects capturing genuine structural differences in TB epidemiology and health system context, rather than representing arbitrary aggregation.

Of the identified surveys, 64 also reported sex-stratified smear-positive TB prevalence. All these studies reported the number of participants, for a total of 2,825,955 (45.6% male). The estimated M:F ratio of smear-positive TB was higher than the bacteriologically confirmed TB estimate for the pooled survey data (2.38; 95% CrI: 1.91, 2.90) and for each world region, when estimated using only those surveys that reported both bacteriologically confirmed and smear-positive TB prevalence (Table N in S1 Appendix). The M:F ratios of smear-positive TB prevalence varied among WHO regions—Americas: 1.26 (95% CrI: 0.51, 2.42), Africa: 1.82 (95% CrI: 1.42, 2.26), Eastern Mediterranean: 2.09 (95% CrI: 1.20, 3.31), Western Pacific: 2.58 (95% CrI: 1.95, 3.33), and South-East Asia: 3.65 (95% CrI: 2.97, 4.38).

**Fig 4:**
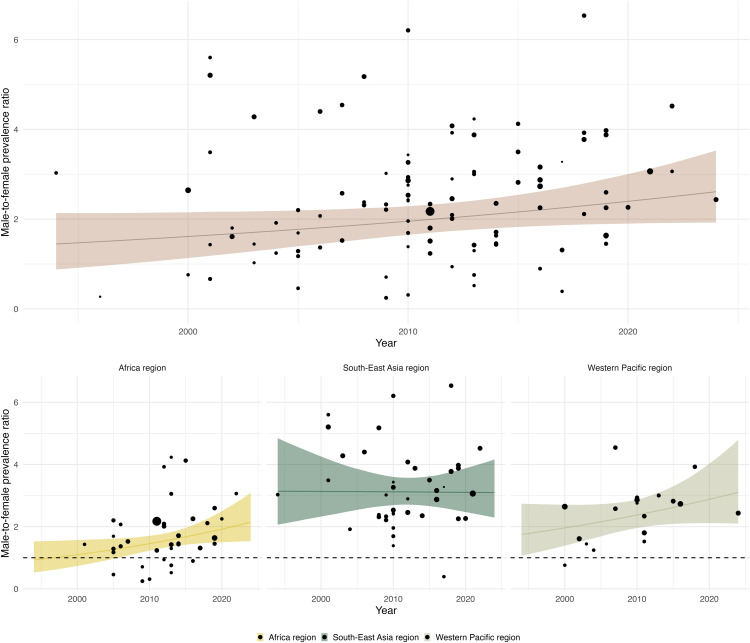
Estimated annual male-to-female ratios of bacteriologically confirmed TB between 1994 and 2020. Panel **A**: All included surveys. Panel **B** and **C**: World regions. Notes: Shaded area represents 95% credible interval. Points on the plot show the empirical male-to-female prevalence ratio for each study, with size corresponding to 1/the standard error of the log odds ratio of male-to-female prevalence, which is used to weight the relative influence of each data point on the regression. Due to the small number of surveys and subsequent uncertainty, we do not present temporal analysis for the Americas (*N* = 2) or Eastern Mediterranean (*N* = 4) regions.

Survey year, defined as the last year of data collection, ranged from 1994 to 2024; five surveys did not report the survey end year (two in Africa, two in South-East Asia, and one in Western Pacific regions). We estimated a likely increasing, but uncertain, time trend in the M:F ratio of bacteriologically confirmed TB (Fig 4). On average, the M:F prevalence ratio increased by 2.0% (95% CrI: −0.2, 4.5%) annually, with a probability of positive direction (PPD) of 0.96. The Africa region had the greatest annual rate of increase, with an average annual change of 2.9% (95% CrI: 0.2, 6.0%) and a PPD of 0.98, while trends in other regions were more uncertain (Table O in S1 Appendix). In exploratory subgroup analyses of 5-year bands, the M:F ratio ranged from 1.68 (95% CrI: 1.01, 2.58) during 2005–2010 to 2.84 (95% CrI: 1.50, 4.64) during 2020–2024; however, these years were among those with the fewest number of prevalence surveys (2005–2010: 18 total, 6 nationally-representative; 2020–2024: 6 total; 2 nationally-representative). We found no evidence that the use of Xpert MTB/RIF or culture or requiring symptoms for sputum collection modified the estimated annual percentage change (Table P and Fig D in S1 Appendix).

In univariable exploratory analyses using data from 38 of 40 nationally-representative surveys, we estimated a positive association between the M:F ratio and GDI (multiplicative effect: 1.15; 95% CrI: 1.03, 1.28; Table 1). In multivariable analysis, the uncertainty in this estimate widened (1.16; 95% CrI: 1.00, 1.34). We also estimated a positive association with excess HIV/AIDS prevalence in men (1.18; 95% CrI: 1.03, 1.35). Together, the included covariates accounted for 60% (95% CrI: 38%, 78%) of the variation observed, as measured by Bayes R^2^. These estimates were robust to prior choice (Table Q in S1 Appendix). We assessed the correlation between covariates and found minimal evidence of strong correlation. The strongest correlation was observed between alcohol use disorder and GDI (0.36; 95% CrI: 0.10, 0.58); Fig E in S1 Appendix presents the estimated covariate correlations. We also estimated M:F ratios for each of the countries included in the multivariable analysis (Table R in S1 Appendix); these ranged from 1.80 (95% CrI: 1.15, 2.69) in Eswatini to 3.79 (95% CrI: 2.48, 5.57) in Viet Nam.

In subgroup analysis, surveys that were nationally representative (*N* = 40), with low risk of bias (*N* = 46), or that did not require symptom screening for sputum collection (*N* = 66) all had M:F ratios of bacteriologically confirmed TB prevalence greater than 2.0 (Table 2). All of the five countries that had five or more surveys—India (*N* = 27), Ethiopia (*N* = 11), China (*N* = 6), Bangladesh (*N* = 5), Viet Nam (*N* = 5)—had mean M:F ratios greater than 1.0, but only India, Bangladesh, and China had M:F ratios for which the 95% credible interval excluded the null (1.0), with 3.28 (95% CrI: 2.68, 3.91), 3.25 (95% CrI: 1.54, 5.57), and 2.65 (95% CrI: 1.77, 3.57) times higher prevalence among males compared to females, respectively. We also found that surveys published after the Horton and colleagues (2016) review had a higher pooled M:F ratio of 2.34 (95% CrI: 1.95, 2.79) compared with those included in the previous review, which had a pooled M:F ratio of 1.79 (95% CrI: 1.39, 2.25).

**Table 2:** Estimated male-to-female ratios of bacteriologically-confirmed TB among key survey subgroups.

	Male-to-female prevalence ratio			Surveys (*N*)	Participants (*N*)	Percentage male (%)
	Posterior mean	Posterior median	95% Credible interval			
Main estimate	2.02	2.01	1.71, 2.34	102	4,658,310	45.55
Nationally representative	2.29	2.29	2.02, 2.58	40	2,077,101	43.35
Subnational	1.51	1.49	1.02, 2.12	62	2,111,659	47.72
Low risk of bias	2.26	2.25	1.93, 2.61	46	2,638,129	45.23
Not low risk of bias	1.76	1.74	1.31, 2.31	56	1,550,631	46.11
Required symptoms for sputum collection	1.45	1.41	0.84, 2.28	36	1,031,361	44.57
Did not require symptoms for sputum collection	2.20	2.19	1.90, 2.52	66	3,148,094	48.58
Required positive culture or Xpert for bacteriologically- confirmed TB diagnosis	2.27	2.26	1.88, 2.72	38	1,358,705	44.13
Did not require positive culture or Xpert for bacteriologically- confirmed TB diagnosis	2.17	2.17	1.77, 2.61	64	2,660,533	46.10
Included in Horton and colleagues 2016 [7]	1.79	1.78	1.39, 2.25	61	2,283,489	46.83
Published after Horton and colleagues 2016 [7]	2.34	2.33	1.95, 2.79	41	1,905,271	44.02
Countries						
Bangladesh	3.25	3.15	1.54, 5.57	5	449,163	45.48
China	2.65	2.64	1.77, 3.57	6	127,688	49.08
Ethiopia	1.13	1.11	0.68, 1.69	11	215,743	49.12
India	3.28	3.27	2.68, 3.91	27	1,619,109	47.50
Viet Nam	2.16	1.98	0.63, 4.77	5	271,526	46.27
5-year bands						
2000–2004	1.97	1.93	1.18, 2.99	14	654,600	47.05
2005–2009	1.68	1.69	1.01, 2.58	19	632,055	47.58
2010–2014	2.02	2.01	1.64, 2.43	39	1,444,720	45.92
2015–2019	2.49	2.48	1.92, 3.17	22	847,303	43.73
2020–2024	2.85	2.79	1.50, 4.64	6	584,673	43.46

Among the 102 surveys included in this analysis, 46, 41, and 14 were assessed to have low, moderate, and high risk of bias, respectively; one survey did not report sufficient information to assess the risk of bias. Of the assessed criteria, surveys were evaluated to have minimal nonresponse bias (51/102 surveys) or presented sufficient information to evaluate study population representativeness (49/102 surveys) less frequently than the other criteria. Fig F in S1 Appendix shows the distribution of studies across bias assessment criteria across the overall bias assessment levels. We found no evidence of publication bias as evaluated via the doi plot and an estimated LFK index of 0.08 (value < |1| indicative of no asymmetry; Fig G in S1 Appendix) [128–130].

## Discussion

This systematic review and meta-analysis synthesized data on more than 4 million participants in 102 community-representative prevalence surveys from five global regions. We found that bacteriologically confirmed TB prevalence was over twice as high among men compared with women in LMICs. Strikingly, 90 of 102 surveys had an estimated TB prevalence among men that exceeded that among women, with a pooled M:F prevalence ratio of 2.02 (95% CrI: 1.71, 2.34). These findings are consistent with other studies, including the previous systematic review we updated in this study [7].

As the identified surveys collected data across a 30-year (1994–2024) period, we were able to analyze the change in M:F prevalence ratios over time. These estimates suggest that male-to-female inequalities in TB prevalence appear to be growing (albeit with substantial uncertainty), even with increased acknowledgement of men’s disproportionate TB burden by WHO and other public health organizations [12,131]. This rising trend was clearer in WHO Africa region, with an estimated 3% annual increase in the M:F prevalence ratio. These trends reflect the persistent (and potentially growing) impact of factors driving sex differences in TB prevalence. Despite strong evidence pointing to excess TB burden among men, and increasing recognition of this excess burden [1,12,13], efforts to address this difference have been insufficient to produce statistically discernable reductions in the M:F ratio of bacteriologically confirmed TB prevalence.

To further investigate the change in the M:F ratio over time, we also analyzed surveys in 5-year subgroups. Of note, while the estimated difference in TB prevalence was among surveys with data collection coinciding with the COVID-19 pandemic (2020–2024), this estimate pooled the results of only six (two nationally-representative) surveys. Therefore, the estimated M:F ratio for this subgroup should be interpreted with caution. The differential impact of the pandemic on TB burden by sex is varied and likely context-specific. A study of TB notifications during the COVID-19 pandemic found evidence supporting differences in the association between missed or delayed diagnoses due to COVID-19, with 22.5% of the 40 examined countries showing a greater impact among women and a similar percentage showing greater impact among men [132].

In further subgroup analyses and alternative model specification, we estimated the sex differences among surveys that required symptoms for sputum collection and those that did not. We found evidence that requiring symptoms was associated with a substantial reduction in the estimated M:F ratio of bacteriologically confirmed TB. This finding suggests that symptom reporting is a sex-differential measurement mechanism. Consequently, approaches to TB detection that require symptom reporting may lead to further under-detection of TB among men. This finding is consistent with previous literature reporting differential symptom-reporting behaviors by sex [133,134].

Improving the uptake of TB prevention and care among men is essential to ending the TB epidemic and ensuring that the global TB response is person-centered and gender-responsive. In exploratory multivariable analyses, we found a significant association between higher male HIV prevalence (relative to females) and higher M:F TB prevalence ratios. This finding suggests that in countries with a higher excess HIV prevalence among men, the M:F TB prevalence ratio might be elevated. On an individual level, HIV increases the risk of progression from *Mycobacterium tuberculosis* infection to TB disease [135–137], the severity of TB disease [138,139], and TB-associated mortality [140,141]. While the majority of people living with HIV now receive anti-retroviral therapy (ART), UNAIDS estimates suggest that men are less likely to know their HIV status, be enrolled in ART or achieve viral suppression [11]. ART is a proven tool in the reduction of TB incidence among people living with HIV [142,143]; therefore, increasing the uptake and continuation among men could reduce TB prevalence in this population as well.

We also estimated a positive association between the M:F prevalence ratio and the GDI. The GDI is calculated as the ratio of a country’s human development index (HDI) among women compared with the HDI among men. A higher value of the GDI indicates lower inequities in health, education, and command of financial resources faced by women. The positive relationship estimated in our analyses suggests greater M:F ratios of bacteriologically confirmed TB prevalence in countries with more equitable gender development. The mechanisms generating this relationship are unclear, and it could be induced by multiple other factors correlated with both GDI distribution and TB risk factors. For this reason, any causal interpretation of these associations is speculative. We leave these questions for future research to further elucidate the drivers of sex differences in TB.

Structural and cultural factors may also contribute to men’s higher TB prevalence beyond the risk factor differences we were able to include in our analysis. Previous research has highlighted how gender norms and expectations influence healthcare access and utilization among men [144]. Men have lower rates of healthcare attendance compared to women, which may be due to perceived stigma, weakness, or lack of control [145] and competing priorities, particularly in settings where men are expected to fulfill provider roles [133]. Adapting the healthcare experience to be gender-responsive and male-friendly may increase TB diagnoses and successful treatment among men [146]. Qualitative studies of male TB survivors, stakeholders, and healthcare workers have identified preferences for modified TB screening interventions that incorporate outreach to male-dominated workplaces and sociocultural settings, as well as the creation of male-only spaces and communication materials [147,148]. These priorities align with WHO’s identification of “men in settings where healthcare access is not tailored to their needs” as a key TB vulnerable population in 2025 [13].

If sex differences in TB prevalence are growing, the interventions aimed at reducing excess TB burden among men have increasing urgency and impact. Community-based interventions have the potential to substantially improve men’s engagement with the TB care cascade. Previous research has investigated the feasibility of screening at occupation centers or key transportation hubs [149–151]. Additional interventions have further attempted to “meet men where they are” through outreach to male-dominated socio-cultural centers, such as bars, betting halls, or churches [152]. Implementing a combination of health facility- and community-based interventions may increase men’s access to diagnosis and treatment, thereby reducing TB burden among men. TB intervention development can be further informed through previous research done to increase men’s engagement in other disease care cascades, such as HIV testing and treatment and diabetes management [153–155]. Evaluation of the implementation of these community-based strategies and developing guidance for male-focused TB screening could be incorporated into strategies to reduce TB incidence in LMICs.

This study has several limitations. The geographic distribution of included surveys was uneven, with over-representation of large, high TB burden countries. While this may bias global generalizability, such concentration arguably reflects an appropriate focus of prevalence surveys on settings with the greatest burden and need. Heterogeneity in survey methods—such as differences in sampling strategies, screening algorithms, and diagnostic tools—introduces additional nonsampling variability that may affect cross-survey comparability. Our definition of bacteriologically confirmed TB was dependent on the methods employed in each survey and could not be fully standardized. To mitigate this, we prioritized test results from more sensitive diagnostics (e.g., Xpert MTB/RIF and culture) when available, although some variation remains. The use of symptom-based screening could also introduce bias in sex-stratified analyses, as the prevalence of TB-related symptoms and their reporting rates may differ by sex, potentially affecting the male-to-female prevalence ratio. We addressed this by attempting to quantify this difference in the included surveys. Furthermore, the estimated M:F ratios of TB prevalence may reflect social and behavioral factors, as discussed above, or an imbalance of demographic or health risk factors, such as age or HIV status, either in the underlying population or survey sample. While we did detect a relationship between excess HIV and TB prevalence among men, our covariate analyses did not account for measurement error in country-level TB determinants, which may have affected coefficient estimates.

As with any systematic review, our estimates may reflect publication and language biases. Our data were restricted to prevalence surveys with publicly available data, either through formal published reports or manuscripts indexed in the electronic databases searched. We did not find evidence of effect size bias in our dataset, suggesting the impact of any publication bias is small. Our data were further limited to only English language studies; to our knowledge, only one prevalence survey was excluded due to this reason. A robustness check found no additional relevant surveys in Spanish, French, or Portuguese. Finally, despite the large overall sample size, uncertainty remains substantial for many of the relationships examined. The 95% credible intervals for several estimates were inclusive of the null relationship or overlapping in subgroup analyses; these findings are directionally consistent with existing evidence but should be interpreted cautiously due to the uncertainty of these estimates.

In summary, we found strong evidence that adult men have over twice the prevalence of pulmonary TB as compared to women in LMICs. These estimated differences in male-to-female bacteriologically confirmed TB prevalence may have increased over time, in particular in WHO Africa region. If sex-related inequalities in TB burden are growing, developing effective strategies to reduce men’s risk of TB and to engage men in TB prevention and care will be essential to end TB.

## Supporting information

S1 Appendix Supplementray Tables A–R and Figures A–G. **Table A**: Full search strings for each database in the systematic review. **Table B**: Inclusion and exclusion criteria for systematic review study selection. **Table C**: Full text review exclusion reasons. **Table D**: Search strings for publications in French, Spanish, and Portuguese as a robustness check of the search strategy. **Fig A**: Decision tree for reported estimates used in meta-analysis of sex-stratified prevalence bacteriological positive TB estimates. **Table E**: Model equations and corresponding priors. **Table F**: Surveys with missing covariate data and the corresponding management approach. **Table G**: Studies excluded at full-text review with their corresponding exclusion reasons. **Table H**: Selected characteristics and reported data of 102 TB prevalence surveys included in quantitative analysis. **Table I**: Cumulative characteristics of study participants in the included prevalence surveys (*N*=102). Note: not all surveys included all reported characteristics. Bacteriologically-confirmed TB count is the sum of reported bacteriologically-confirmed case counts and case counts estimated from reported prevalence risks and survey participants. Of 102 surveys, 82 reported case counts, 16 were estimated, and 4 were missing the information to estimate case counts. Similarly, among 64 surveys reporting smear-positive TB prevalence, 54 reported case counts and 10 surveys had case counts estimated. Culture-positive TB case counts were reported by 27 surveys. **Table J**: Model performance statistics. **Fig B**: Density and trace plots for selected model fits. Model 1: i. Main effect. Model 2: ii. Main effect across world regions. Model 3: iii. Univariable analysis of the study end year on the main effect (fit to all surveys). Model 4: iv. Univariable analysis of study end year on main effect across world regions (fit to all surveys). Model 35: v. Multi-variable analysis of covariates on main effect. Model 36: vi. Main effect fit to smear-positive TB prevalence ratios. Model 37: vii. Main effect across world regions fit to smear-positive TB prevalence ratios. **Table K**: Male-to-female ratio of bacteriologically-confirmed TB prevalence as estimated by alternative model hierarchical structures. Model descriptions: 1. Main model specification with country- and survey-level random effects. 2. Alternative model with country-level random effects nested in WHO region random-effects and survey-level random effects. **Table L**: Estimated impact of screening and diagnostic algorithms on the male-to-female ratio of bacteriologically-confirmed TB prevalence. Note: coefficients are reported on the natural log-scale. Model descriptions: 1. Main effects model with country- and survey-level random effects. 2. Main model with an additional binary variable describing whether a survey required symptoms for sputum collection. 3. Main model with an additional binary variable describing whether Xpert or culture was required for a bacteriologically confirmed diagnosis covariate. That is, an individual must receive an Xpert or culture test and at least one of these must be positive for a bacteriologically-confirmed TB diagnosis. 4. Main model with an additional binary variable describing whether Xpert and/or culture testing were conducted. That is, the survey used Xpert and/or culture (in addition to or instead of sputum smear microscopy), and a positive result on any of these tests was sufficient for bacteriologically-confirmed TB diagnosis. **Fig C**: Posterior heterogeneity across main effect models with country or WHO region random effects. **Table M**: Posterior summary of variance in main effect models with country or WHO region random effects. Note: World Health Organization (WHO). **Table N**: Male-to-female prevalence ratio estimates for bacteriologically-confirmed TB and smear-positive TB by world region. Notes: Bacteriologically-confirmed (BC); smear-positive (SP). Estimates represent the posterior mean (posterior median; 95% credible interval). **Table O**: Estimated annual percentage change in male-to-female ratios of bacteriologically-confirmed TB prevalence by world region. **Table P**: Estimated effect of screening and diagnostic algorithms on the temporal model of male-to-female ratio of bacteriologically confirmed TB prevalence. Note: coefficients are reported on the natural log-scale. Model descriptions: 1. Main temporal model specification with survey- and country-level random effects. 2. Main temporal model with an additional binary variable describing whether a survey required symptoms for sputum collection. 3. Main temporal model with an additional binary variable describing whether a survey required symptoms for sputum collection and accounts for an interaction between this variable and the study end year. 4. Main temporal model with an additional binary variable describing whether Xpert or culture was required for a bacteriologically confirmed diagnosis. That is, an individual must receive an Xpert or culture test and at least one of these be positive for a bacteriologically-confirmed TB diagnosis. 5. Main temporal model with an additional binary variable describing whether Xpert or culture was required for a bacteriologically confirmed diagnosis and accounts for an interaction between this variable and study end year. 6. Main temporal model with an additional binary variable describing whether Xpert and/or culture testing were available. That is, the survey used Xpert and/or culture (in addition to or instead of sputum smear microscopy), and a positive result on any of these tests was sufficient for bacteriologically-confirmed TB diagnosis. 7. Main temporal model with an additional binary variable describing whether Xpert and/or culture testing were conducted and accounts for an interaction between this variable and study end year. **Fig D**: Trends in the male-to-female ratio of bacteriologically-confirmed TB prevalence as estimated by alternative model specifications. i. Symptoms required versus symptoms not required. ii. Xpert or culture is required versus not required for bacteriologically-confirmed TB diagnosis. iii. Xpert or culture used versus not used for bacteriologically-confirmed TB diagnostic pathway. Model descriptions: i. Main model with a binary variable for whether symptoms were required for sputum collection. Accounts for an interaction with survey end year. ii. Main model with a binary variable for whether Xpert or culture is required for bacteriologically confirmed diagnosis covariate. That is, an individual must receive an Xpert or culture test and at least one of these is required for a bacteriologically-confirmed TB diagnosis. Accounts for an interaction with the survey end year. iii. Main model with a binary variable for Xpert or culture used for diagnosis covariate. That is, an individual must receive an Xpert or culture test, but a smear-positive test was sufficient for a bacteriologically-confirmed TB diagnosis. Accounts for an interaction with survey end year. **Fig E**: Posterior distribution of the correlation of model covariates. Note: the dot represents the mean of the posterior distribution, and the black line represents the 95% credible interval. **Table Q**: Estimated impact of alternative priors on the covariate model of the male-to-female ratio of bacteriologically-confirmed TB prevalence. Note: Coefficients are reported on the natural log-scale. Model descriptions: All models have the same model specification, which includes study- and survey-level random effects. These models are all fit to nationally-representative survey data. Models differ only in their prior on the covariate regression coefficients: 1. Normal distribution with mean = 0 and standard deviation = 1. 2. Normal distribution with mean = 0 and standard deviation = 10. 3. Cauchy distribution with location = 0 and scale = 1.25. **Table R**: Country-level estimates of male-to-female ratios of bacteriologically-confirmed TB prevalence as calculated from the multivariate regression model. **Fig F**: Distribution of overall risk of bias by each assessed criterion. **Fig G**: Doi plot to evaluate potential publication bias among included prevalence surveys. Note: The figure above is used to examine symmetry across effect sizes. This symmetry is quantified through the LFK index, where | LFK value < 1 indicates no evidence of asymmetry.
(DOCX)

S1 Form Extraction form for systematic review of tuberculosis prevalence in low- and middle-income countries. (DOCX)

S1 Checklist MOOSE checklist. (DOCX)

S2 Checklist PRISMA 2020 checklist (with PRISMA 2020 Abstract checklist). *From:* Page MJ, McKenzie JE, Bossuyt PM, Boutron I, Hoffmann TC, Mulrow CD, and colleagues. The PRISMA 2020 statement: an updated guideline for reporting systematic reviews. *MetaArXiv*. 2020, September 14. https://doi.org/10.31222/osf.io/v7gm2. For more information, visit: www.prisma-statement.org.
(DOCX)

## Abbreviations

AT: anti-retroviral therapy
BMI: body mass index
CrI: credible interval
EAPC: estimated annual percentage change
ESS: effective sample size
GBD: Global Burden of Diseases
GDI: Gender Development Index
HDI: Human Development Index
LMICs: low- and middle-income countries
M:F: male-to-female
MOOSE: Meta-analysis of Observational Studies in Epidemiology
NMT: neural machine translation
PPD: probability of positive direction
PRISMA: Preferred Reporting Items for Systematic Reviews and Meta-analyses
PROSPERO: International Prospective Register of Systematic Reviews
TB: tuberculosis
WHO: World Health Organization.
