# Supplementary material for: Differences in tuberculosis prevalence by sex in low- and middle-income countries over 1993–2025: A systematic review and meta-analysis
Source: PLoS Med. 2026 May 22;23(5):e1005114. doi: 10.1371/journal.pmed.1005114 (PMC13225659; doi:10.1371/journal.pmed.1005114)
Supplement: S1 Form — (DOCX) [file pmed.1005114.s002.docx]

**S1 Form: Extraction form for “Differences in tuberculosis prevalence by sex in low- and middle-income countries over 1993-2025: a systematic review and meta-analysis”**

**1. Study identification**

**1.1. Study DOI:** Please input only the unique DOI and not the full URL. 
 For example, "10.1371/journal.pgph.0000784". 
 If extracting data from multiple publications, input the primary prevalence survey DOI first,
 followed by the DOI for each additional paper, separated by a semi-colon. Only include
 those papers from which you have extracted data.
 Write NA if not provided.

**1.2. First Author's surname:**

**1.3. Paper Title:**

**1.4. Publication Year:**

**2. Corresponding Author**

**2.1. Corresponding author (surname, initials):**

**2.2. Corresponding author's email address:** Record phone number or mailing address if no email address is provided.

**3. Study methodology** If both adults and children were included in the study population, answer the following
 questions regarding methods used for adult participants.

**3.1. Does the study report that it has adhered to guidelines from the WHO handbook for prevalence surveys?**

1. **Yes**
2. **No**
3. **Unknown**

**3.2. Selection of study population**

1. **Recruitment of all individuals within one or more areas**
2. **Simple random sampling within one cluster**
3. **Simple random sampling within >1 cluster**
4. **Multistage random sampling within >1 cluster (with or without weighting)**

**3.2.1. Does this study include multiple prevalence surveys?**

1. **No**
2. **Yes (If yes, you will be directed to another page to fill in requisite details)**
3. **Unsure (requires discussion with team)**

**3.3. Country(ies) of study:** If multiple countries, separate each with a comma and a space

**3.4. Study Setting:** Provide any contextual / geographical details of where the study was conducted.

**3.5. What was the geographic coverage of the study?**

1. **Single area (e.g., province, district, city)**
2. **Multiple areas (but not nationally representative)**
3. **Nationally representative**
4. **Other**

**3.6 What was the urban/rural coverage of the study?**

1. **Urban only**
2. **Rural only**
3. **Urban and rural**
4. **Unknown**

**3.7. Study Year(s) (time-period during which study was conducted):** Record four-digit year; if multiple years then use format ‘9999-9999.’

**3.8. Were contact investigations used to identify additional participants?** Make sure to exclude mass-surveys around circumference of homes.

1. **Yes**
2. **No**
3. **Unknown**

**4. Screening**

**4.1. Screening criteria** Description (optional)

**4.2. Were individuals currently on TB treatment included in the study population?**

1. **Yes**
2. **No**
3. **Unknown**

**4.3. Were participants screened prior to diagnosis?**

1. **Yes**
2. **No**

**4.4. Which of the following screening tests were done? (Select those that apply.)**

Example for "Other": Latent TB test

1. **Chest X-ray only**
2. **Symptom screen only**
3. **Symptom screen, then chest X-ray (if positive)**
4. **Chest X-ray, then symptom screen (if positive)**
5. **Chest X-ray and symptom screen**
6. **No screening performed**
7. **Other**

**4.5. Answer if symptom screen was performed. Symptom screen positive defined by:**

**(Select those that apply.)**

1. **Cough ≥ 3 weeks**
2. **Cough ≥ 2 weeks**
3. **Cough of other or unknown duration**
4. **Sputum production**
5. **Haemoptysis**
6. **Chest pain**
7. **Fever**
8. **Night sweats**
9. **Weight loss**
10. **Symptom screen was not performed**
11. **Other**

**4.6. If chest X-ray was used, how was a positive result defined?**

1. **Any abnormality**
2. **Indicative of TB**
3. **Chest X-ray was not used**
4. **Other**

**5. Diagnostics**

**5.1. From which participants was sputum collected?**

1. **All participants**
2. **Participants with symptoms**
3. **Participants with abnormal chest X-ray**
4. **Participants with symptoms OR abnormal chest X-ray**
5. **No sputum collected**
6. **Unknown**
7. **Other**

**5.2. Were sputum samples tested by smear microscopy?**

1. **Yes**
2. **No**
3. **No sputum collected**
4. **Unknown**

**5.3. Which samples were tested by smear microscopy?**

1. **All samples**
2. **Subsets of samples (Specify in 5.3.1)**
3. **No samples tested by smear microscopy**
4. **Unknown**
5. **Other**

**5.3.1. If "Subsets of samples" was selected in 5.3, specify:**

**5.4. Which microscopy method was used?**

1. **Light microscopy**
2. **Fluorescence**
3. **Light and Fluorescence microscopy**
4. **No samples tested by smear microscopy**
5. **Unknown**
6. **Other**

**5.5. Were samples tested by Xpert MTB/RIF, or Xpert Ultra?**

1. **Yes (Xpert MTB/RIF only)**
2. **Yes (Xpert Ultra only)**
3. **Yes (both Xpert MTB/RIF and Xpert Ultra, or either of these)**
4. **Neither**
5. **Unknown**

**5.6. Which samples were tested by Xpert?**

1. **All samples**
2. **Subset of samples (Specify in 5.6.1)**
3. **No samples tested by Xpert**
4. **Unknown**
5. **Other**

**5.6.1. If "Subset of samples" was selected in 5.6, specify:**

**5.6.2. Were Xpert trace results classified as "Microbiologically-confirmed TB"?**

1. **Yes**
2. **No**
3. **No samples tested by Xpert**

**5.7. Were sputum samples tested by culture?**

1. **Yes**
2. **No**
3. **Unknown**

**5.8. Which samples were tested by culture?**

1. **All samples**
2. **Subset of samples (Specify in 5.8.1)**
3. **No samples tested by culture**
4. **Unknown**
5. **Other**

**5.8.1. If "Subset of samples" was selected in 5.8, specify:**

**5.9. Which culture media was used?**

1. **Solid**
2. **Liquid**
3. **Solid and liquid**
4. **No samples tested by culture**
5. **Unknown**

**5.9.1 If none of the above, which test was used?**

**6. Case Definitions** Give explanations for any case definitions used in the study. Write "N/A" for those that are
 not used in the study.

**6.1. Presumptive TB (may be called "suspect TB" or "TB suspect" or "possible TB case", or similar in some reports)**

**6.2. Radiologically-confirmed TB**

**6.3. Bacteriologically-confirmed TB (may be called "microbiologically-confirmed TB", or similar in some reports)**

**6.4. Culture-positive TB**

**6.5. Sputum smear-positive TB**

**6.6. Sputum smear-negative TB**

**6.7. TB without bacteriological confirmation**

**6.8. Prevalent TB (please specify if treatment was provided or not)**

**6.9. If given, provide the definition of Rural vs. Urban participants**

**6.10. If given, provide the definition for how HIV status was ascertained.**

1. Self-report
2. HIV test
3. Not specified
4. HIV status was not assessed

**6.11. Any other case definition(s)**

**6.12. Additional comments (optional)**

**6.13. Were prevalence estimates adjusted in some way (synonyms: “standardized”, “weighted”, “controlled”)? If so, describe how. If not, enter, “no”.**

**6.14. Additional comments on study methodology**

**7. Results**If both adults and children were included in the study population, answer the following questions regarding adults only.

**7.1. Participant description (optional)**

**7.2. What is the total target population of the survey area(s)?**

Enter a number.

**7.3. Does this study report results by sex?** If no, fill out the total column and leave other cells blank. Please write "N/A" if a cell in the "total" column
 is unreported**.**If yes, please fill out table 7.3.1 completely, entering "N/A" if a value is not reported.

1. **Yes**
2. **No**

**7.3.1 Results (by sex)** If a data cell is not reported, please type "N/A".

|  | **Male** | **Female** | **Total** |
| --- | --- | --- | --- |
| **7.3.1. Eligible participants** |  |  |  |
| **7.3.2. Participants** |  |  |  |
| **7.3.3. People with presumptive TB** |  |  |  |
| **7.3.4. People with TB symptoms** |  |  |  |
| **7.3.5. People with abnormal chest X-ray** |  |  |  |
| **7.3.6. Persons with successful sputum samples** |  |  |  |
| **7.3.7. Radiologically-confirmed TB [bacteriological unconfirmed]** |  |  |  |
| **7.3.8. Bacteriologically-confirmed TB** |  |  |  |
| **7.3.9. Sputum smear-positive TB** |  |  |  |
| **7.3.10. Culture-positive TB** |  |  |  |
| **7.3.11. Prevalent TB** |  |  |  |
| **7.3.12. [Crude] Prevalence of bacteriologically-confirmed TB (per 100,000)** |  |  |  |
| **7.3.13. [Crude] Confidence Interval of 7.3.12** |  |  |  |
| **7.3.14. [Crude] Prevalence of sputum smear-positive TB (per 100,000)** |  |  |  |
| **7.3.15. [Crude] Confidence Interval of 7.3.14** |  |  |  |
| **7.3.16. [Crude] Prevalence of all forms of TB (per 100,000)** |  |  |  |
| **7.3.17. [Crude] Confidence Interval of 7.3.16** |  |  |  |
| **7.3.18. [Adjusted] Prevalence of bacteriologically-confirmed TB (per 100,000)** |  |  |  |
| **7.3.19. [Adjusted] Confidence Interval of 7.3.18** |  |  |  |
| **7.3.20 [Adjusted] Prevalence of sputum smear-positive TB (per 100,000)** |  |  |  |
| **7.3.21 [Adjusted] Confidence Interval of 7.3.20** |  |  |  |
| **7.3.22 [Adjusted] Prevalence of all forms of TB (per 100,000)** |  |  |  |
| **7.3.23 [Adjusted] Confidence Interval of 7.3.22** |  |  |  |

**7.4 Does this study report results by urban/rural stratification?** If no, leave all cells blank and move to 7.5.

If yes, please fill out table 7.4.1 completely, entering "N/A" if a value is not reported.

1. **Yes**
2. **No**

**7.4.1 Results (by rurality)** If a data cell is not reported, please type "N/A".

|  | **Urban** | **Rural** | **Total** |
| --- | --- | --- | --- |
| **7.4.1. Eligible participants** |  |  |  |
| **7.4.2. Actual participants** |  |  |  |
| **7.4.3. People with presumptive TB** |  |  |  |
| **7.4.4. People with TB symptoms** |  |  |  |
| **7.4.5. People with abnormal chest X-ray** |  |  |  |
| **7.4.6. Persons with successful sputum samples** |  |  |  |
| **7.4.7. Radiologically-confirmed TB [bacteriological unconfirmed]** |  |  |  |
| **7.4.8. Bacteriologically-confirmed TB** |  |  |  |
| **7.4.9. Sputum smear-positive TB** |  |  |  |
| **7.4.10. Culture-positive TB** |  |  |  |
| **7.4.11. Prevalent TB** |  |  |  |
| **7.4.12. [Crude] Prevalence of bacteriologically-confirmed TB (per 100,000)** |  |  |  |
| **7.4.13. [Crude] Confidence Interval of 7.4.12** |  |  |  |
| **7.4.14. [Crude] Prevalence of sputum smear-positive TB (per 100,000)** |  |  |  |
| **7.4.15. [Crude] Confidence Interval of 7.4.14** |  |  |  |
| **7.4.16. [Crude] Prevalence of all forms of TB (per 100,000)** |  |  |  |
| **7.4.17. [Crude] Confidence Interval of 7.4.16** |  |  |  |
| **7.4.18. [Adjusted] Prevalence of bacteriologically-confirmed TB (per 100,000)** |  |  |  |
| **7.4.19. [Adjusted] Confidence Interval of 7.4.18** |  |  |  |
| **7.4.20. [Adjusted] Prevalence of sputum smear-positive TB (per 100,000)** |  |  |  |
| **7.4.21. [Adjusted] Confidence Interval of 7.4.20** |  |  |  |
| **7.4.22 [Adjusted] Prevalence of all forms of TB (per 100,000)** |  |  |  |
| **7.4.23. [Adjusted] Confidence Interval of 7.4.22** |  |  |  |

**7.5 Does this survey/study report results by HIV status?**

If no, leave all cells blank and move to 7.6.

If yes, please fill out table 7.5.1 completely, entering "N/A" if a value is not reported.

1. Yes
2. No

**7.5.1 Results (by HIV status)**If a data cell is not reported, please type "N/A".

|  | **HIV-positive** | **HIV-negative** | **Total** |
| --- | --- | --- | --- |
| **7.5.1. Eligible participants** |  |  |  |
| **7.5.2. Participants** |  |  |  |
| **7.5.3. People with presumptive TB** |  |  |  |
| **7.5.4. People with TB symptoms** |  |  |  |
| **7.5.5. People with abnormal chest X-ray** |  |  |  |
| **7.5.6. Persons with successful sputum samples** |  |  |  |
| **7.5.7. Radiologically-confirmed TB [bacteriological unconfirmed]** |  |  |  |
| **7.5.8. Bacteriologically-confirmed TB** |  |  |  |
| **7.5.9. Sputum smear-positive TB** |  |  |  |
| **7.5.10. Culture-positive TB** |  |  |  |
| **7.5.11. Prevalent TB** |  |  |  |
| **7.5.12. [Crude] Prevalence of bacteriologically-confirmed TB (per 100,000)** |  |  |  |
| **7.5.13. [Crude] Confidence Interval of 7.5.12** |  |  |  |
| **7.5.14. [Crude] Prevalence of sputum smear-positive TB (per 100,000)** |  |  |  |
| **7.5.15. [Crude] Confidence Interval of 7.5.14** |  |  |  |
| **7.5.16. [Crude] Prevalence of all forms of TB (per 100,000)** |  |  |  |
| **7.5.17. [Crude] Confidence Interval of 7.5.16** |  |  |  |
| **7.5.18. [Adjusted] Prevalence of bacteriologically-confirmed TB (per 100,000)** |  |  |  |
| **7.5.19. [Adjusted] Confidence Interval of 7.5.18** |  |  |  |
| **7.5.20. [Adjusted] Prevalence of sputum smear-positive TB (per 100,000)** |  |  |  |
| **7.5.21. [Adjusted] Confidence Interval of 7.5.20** |  |  |  |
| **7.5.22 [Adjusted] Prevalence of all forms of TB (per 100,000)** |  |  |  |
| **7.5.23. [Adjusted] Confidence Interval of 7.5.22** |  |  |  |

**7.6 Does this survey/study report results by age group?**

If no, leave all cells blank and move to 7.7.

If yes, please fill out table 7.6.1 completely, entering "N/A" if a value is not reported.

1. Yes
2. No

**7.6.1 Results (by age group)** If a data cell is not reported, please type "N/A".

|  | **0-14** | **15-24** | **25-34** | **35-44** | **45-54** | **55-64** | **65+** | **Total** |
| --- | --- | --- | --- | --- | --- | --- | --- | --- |
| **7.6.1. Age-group definitions, if different (format "XX-YY")** |  |  |  |  |  |  |  |  |
| **7.6.2. Eligible participants** |  |  |  |  |  |  |  |  |
| **7.6.3. Participants** |  |  |  |  |  |  |  |  |
| **7.6.4. People with presumptive TB** |  |  |  |  |  |  |  |  |
| **7.6.5. People with TB symptoms** |  |  |  |  |  |  |  |  |
| **7.6.6. People with abnormal chest X-ray** |  |  |  |  |  |  |  |  |
| **7.6.7. Persons with successful sputum samples** |  |  |  |  |  |  |  |  |
| **7.6.8. Radiologically-confirmed TB [bacteriological unconfirmed]** |  |  |  |  |  |  |  |  |
| **7.6.9. Bacteriologically-confirmed TB** |  |  |  |  |  |  |  |  |
| **7.6.10. Sputum smear-positive TB** |  |  |  |  |  |  |  |  |
| **7.6.11. Culture-positive TB** |  |  |  |  |  |  |  |  |
| **7.6.12. Prevalent TB** |  |  |  |  |  |  |  |  |
| **7.6.13. [Crude] Prevalence of bacteriologically-confirmed TB (per 100,000)** |  |  |  |  |  |  |  |  |
| **7.6.14. [Crude] Confidence Interval of 7.6.13** |  |  |  |  |  |  |  |  |
| **7.6.15. [Crude] Prevalence of sputum smear-positive TB (per 100,000)** |  |  |  |  |  |  |  |  |
| **7.6.16. [Crude] Confidence Interval of 7.6.15** |  |  |  |  |  |  |  |  |
| **7.6.17. [Crude] Prevalence of all forms of TB (per 100,000)** |  |  |  |  |  |  |  |  |
| **7.6.18. [Crude] Confidence Interval of 7.6.17** |  |  |  |  |  |  |  |  |
| **7.6.19. [Adjusted] Prevalence of bacteriologically-confirmed TB (per 100,000)** |  |  |  |  |  |  |  |  |
| **7.6.20. [Adjusted] Confidence Interval of 7.6.19** |  |  |  |  |  |  |  |  |
| **7.6.21. [Adjusted] Prevalence of sputum smear-positive TB (per 100,000)** |  |  |  |  |  |  |  |  |
| **7.6.22. [Adjusted] Confidence Interval of 7.6.21** |  |  |  |  |  |  |  |  |
| **7.6.23 [Adjusted] Prevalence of all forms of TB (per 100,000)** |  |  |  |  |  |  |  |  |
| **7.6.24. [Adjusted] Confidence Interval of 7.6.23** |  |  |  |  |  |  |  |  |

**7.7 Does this study report results by both sex and urban/rural stratification?**

If no, leave all cells blank and move to 7.8.

If yes, please fill out table 7.7.1 completely, entering "N/A" if a value is not reported.

1. Yes
2. No

**7.7.1 Results (by sex and rurality)**

If a data cell is not reported, please type "N/A".

|  | **Male - Urban** | **Female - Urban** | **Male - Rural** | **Female - Rural** | **Total** |
| --- | --- | --- | --- | --- | --- |
| **7.7.1. Eligible participants** |  |  |  |  |  |
| **7.7.2. Participants** |  |  |  |  |  |
| **7.7.3. People with presumptive TB** |  |  |  |  |  |
| **7.7.4. People with TB symptoms** |  |  |  |  |  |
| **7.7.5. People with abnormal chest X-ray** |  |  |  |  |  |
| **7.7.6. Persons with successful sputum samples** |  |  |  |  |  |
| **7.7.7. Radiologically-confirmed TB [bacteriological unconfirmed]** |  |  |  |  |  |
| **7.7.8. Bacteriologically-confirmed TB** |  |  |  |  |  |
| **7.7.9. Sputum smear-positive TB** |  |  |  |  |  |
| **7.7.10. Culture-positive TB** |  |  |  |  |  |
| **7.7.11. Prevalent TB** |  |  |  |  |  |
| **7.7.12. [Crude] Prevalence of bacteriologically-confirmed TB (per 100,000)** |  |  |  |  |  |
| **7.7.13. [Crude] Confidence Interval of 7.7.12** |  |  |  |  |  |
| **7.7.14. [Crude] Prevalence of sputum smear-positive TB (per 100,000)** |  |  |  |  |  |
| **7.7.15. [Crude] Confidence Interval of 7.7.14** |  |  |  |  |  |
| **7.7.16. [Crude] Prevalence of all forms of TB (per 100,000)** |  |  |  |  |  |
| **7.7.17. [Crude] Confidence Interval of 7.7.16** |  |  |  |  |  |
| **7.7.18. [Adjusted] Prevalence of bacteriologically-confirmed TB (per 100,000)** |  |  |  |  |  |
| **7.7.19. [Adjusted] Confidence Interval of 7.7.18** |  |  |  |  |  |
| **7.7.20. [Adjusted] Prevalence of sputum smear-positive TB (per 100,000)** |  |  |  |  |  |
| **7.7.21. [Adjusted] Confidence Interval of 7.7.20** |  |  |  |  |  |
| **7.7.22 [Adjusted] Prevalence of all forms of TB (per 100,000)** |  |  |  |  |  |
| **7.7.23. [Adjusted] Confidence Interval of 7.7.22** |  |  |  |  |  |

**7.8. Additional comments on results and/or data availability.**

**7.9. List any additional references that should be examined for inclusion in this systematic review.**Copy the full citation from the study's reference list; skip a line between each reference.

**8. Study Quality**See Hoy et al. (2012) Appendix for examples and additional details on study quality assessment.

**8.1. Was the study’s sample population a true or close representation of the target population in relation to relevant variables, e.g., age, sex, occupation?**

1. **Yes (low risk)**
2. **No (high risk)**
3. **Unknown**

**8.2. Was some form of random selection used to select the sample or was a census undertaken?**

1. **Yes (low risk)**
2. **No (high risk)**

**8.3. Was the likelihood of non-response bias minimal?**

1. **Yes (low risk)**
2. **No (high risk)**

**8.4. Were data collected directly from subjects (as opposed to a proxy)?**

1. **Yes (low risk)**
2. **No (high risk)**

**8.5. Was an acceptable case definition used in the study?**

1. **Yes (low risk)**
2. **No (high risk)**

**8.6. Was the study instrument that measured the parameter of interest shown to have reliability and validity (i.e., diagnostic methods)?**

1. **Yes (low risk)**
2. **No (high risk)**

**8.7. Was the same mode of data collection used for all subjects?**

1. **Yes (low risk)**
2. **No (high risk)**

**8.8. Were the numerator and denominator for the parameter of interest appropriate?**

1. **Yes (low risk)**
2. **No (high risk)**

**8.9. Summary item on the overall risk of bias (based on 8.1-8.8)**

1. **Low risk of bias - Further research is very unlikely to change our confidence in the estimate**
2. **Moderate risk of bias - Further research is likely to have an important impact on our confidence in the estimates**
3. **High risk of bias - Further research is very likely to have an important impact on our confidence in the estimates**

**8.10. Additional comments on study quality**
